# Medication Adherence, Stigma, and Access-to-Care as Predictors of Psychiatric Relapses in Tunisia

**DOI:** 10.1192/j.eurpsy.2026.12201

**Published:** 2026-07-31

**Authors:** S. Ben Othman, M. Sehli, M. Touzeri, H. Mami, A. Aissa

**Affiliations:** Taher Maamouri Hospital, Nabeul, Tunisia

## Abstract

**Introduction:**

Medication non-adherence and social stigma remain major barriers to relapse prevention in severe mental disorders. In low-resource settings, additional structural obstacles—such as cost and access to care—further compromise treatment continuity. Exploring these factors within the Tunisian context may provide insights into improving relapse prevention strategies.

**Objectives:**

To evaluate how medication adherence, insight, internalized stigma, and structural barriers to care affect psychiatric relapses in Tunisian patients with severe mental disorders

**Methods:**

Eighty-seven hospitalized patients (43 BD, 44 SCZ) were assessed using the Drug Attitude Inventory (DAI-10) for adherence, Birchwood Insight Scale (BIS) for insight, Internalized Stigma of Mental Illness scale (ISMI), and a questionnaire on access-to-care barriers (transport, availability, cost). Outcomes included number of relapses in the previous year. Analyses used Chi-square tests and Spearman correlations.

**Results:**

Non-adherence was observed in 78% of patients (BD: 81%, SCZ: 75%; p=0.47). Insight (BIS mean 35/60) did not correlate with relapse (r=–0.12, p=0.24) or adherence (r=0.09, p=0.38). Internalized stigma (ISMI mean 2.8/4) correlated positively with relapse (r=0.32, p=0.005) and negatively with adherence (r=–0.28, p=0.012). Significant access-to-care barriers associated with relapse included medication shortages (52%; p=0.037) and transport difficulties (22%; p=0.027), particularly in rural areas.

**Conclusions:**

Medication adherence, internalized stigma, and structural barriers significantly influence psychiatric relapses, while insight alone is not predictive. Interventions should combine psychoeducation, anti-stigma programs, family involvement, and improved rural access to care.

**Disclosure of Interest:**

None Declared

